# Retinoic acid regulates donor myeloid-derived suppressor cell and colonic dendrite cell reconstitution after allogeneic hematopoietic stem cell transplantation in mice

**DOI:** 10.3389/fimmu.2026.1599346

**Published:** 2026-09-22

**Authors:** Xinlei Li, Jianwei Zheng, Dian Zhou, Rachel Limpert, Brian Taylor, Linlu Tian, Xue-Zhong Yu, William R. Drobyski, Xiao Chen

**Affiliations:** 1 Division of Hematology & Oncology, Medical College of Wisconsin, Milwaukee, WI, United States; 2 Department of Medicine, Medical College of Wisconsin, Milwaukee, WI, United States; 3 Department of Microbiology and Immunology, Medical College of Wisconsin, Milwaukee, WI, United States

**Keywords:** CD103, dendritic cells, graft-versus-host disease, myeloid derived suppressor cells, retinoic acid

## Abstract

Graft-versus-host disease (GVHD) remains a major obstacle to the success of allogeneic hematopoietic stem cell transplantation (HSCT). Donor hematopoietic progenitor cells reconstitute the recipient’s immune system after transplant, and newly generated donor-derived immune cells actively participate in propagating alloimmunity. Retinoic acid (RA) has well-known effects on the differentiation and proliferation of myeloid progenitor cells. We hypothesized that RA influences the reconstitution of some donor-derived myeloid cell populations after allogeneic HSCT. In a C57BL/6 into Balb/c murine allogeneic bone marrow transplantation (BMT) model, we found that Gr1^+^CD11b^+^CD84^+^F4-80^-^ myeloid-derived suppressor cells (MDSCs) accumulated in the spleen of recipient mice early after BMT. Notably, the absolute number of MDSCs was significantly reduced in RA-treated mice compared with DMSO-treated mice. Furthermore, MDSCs isolated from RA-treated recipient mice showed decreased immunosuppressive function in mixed lymphocyte reactions (MLRs). Bulk RNA-sequencing (RNA-seq) revealed that RA treatment alters transcriptional programs involving Wnt signaling and the CCL2-CCR2 axis in MDSCs. We found that adding RA to the MDSC culture promotes the differentiation of Ly6C^-^Ly6G^+^ granulocytic MDSCs. Thus, enhancing RA signaling causes both quantitative and qualitative changes of MDSCs after allogeneic HSCT. Recent studies have demonstrated an important role of donor intestinal CD103^+^ dendrite cells (DCs) in propagating GVHD. We found that RA-treated recipient mice had significantly increased colonic CD103^+^ DCs compared with DMSO-treated mice. On the contrary, vitamin A deficiency was associated with a significant reduction in the percentage and absolute number of CD103^+^ DCs. To study the effects of RA on DCs *in vitro*, BM cells were cultured in the presence of GM-CSF. There was a significant increase in the percentage of CD11c^+^CD103^+^ mucosal DCs in cultures exposed to RA. Furthermore, RA-exposed DCs exhibited increased MHC-II expression and enhanced T-cell stimulatory capacity. In summary, our study demonstrated that RA participates in regulating donor MDSC and colonic CD103^+^ DC reconstitution after allogeneic HSCT. Therapeutic modulation of RA signaling or its downstream pathways may represent a strategy to limit alloimmunity and improve transplant outcomes.

## Introduction

Graft-versus-host disease (GVHD) remains a major obstacle to broader clinical application of allogeneic hematopoietic stem cell transplantation (HSCT), an effective treatment for many malignant and nonmalignant hematological disorders (1–4). Donor hematopoietic stem/progenitor cells reconstitute the recipient’s immune system after transplant and donor-derived immune cells are critically involved in GVHD pathogenesis (5). It is well established that mature donor T cells are the primary effector cells that cause GVHD. In addition, several types of donor innate immune cells have been shown to be important players in propagating alloimmunity (6). Previous studies have demonstrated that donor antigen presenting cells (APCs) can amplify local tissue damage, thus contributing to GVHD progression (7, 8). Importantly, donor colonic CD103^+^ dendrite cells (DCs) have been shown to play an important role in determining the severity of intestinal and systemic GVHD (9, 10). Recent mechanistic studies have further defined the pathogenic roles of donor intestinal conventional DC subsets in GVHD. Takahashi et al. demonstrated that donor XCR1^+^ conventional DCs and CD11b^+^ conventional DCs differentially promote Th1- and Th17-dependent gastrointestinal GVHD, respectively (11). Besides mediating a detrimental effect, donor innate immune cells may also play an immunoregulatory role (12). Specifically, myeloid-derived suppressor cells (MDSCs) have been implicated in GVHD pathogenesis (13). MDSCs are immature myeloid cells characterized by their ability to suppress T cell proliferation (14, 15). They are found to undergo dramatic expansion in tumor-bearing hosts with various cancers and the accumulation of MDSCs correlates with poor therapeutic responses and clinical outcomes (15–23). Interestingly, accumulation of MDSCs has also been reported in other pathologic conditions such as infectious diseases and traumatic stress (24–29). We and others have shown that MDSCs accumulate in recipient mice after allogeneic bone marrow transplantation (BMT) (30–32). Furthermore, adoptive transfer of *in vitro* generated MDSCs can reduce GVHD severity (33–37). These data suggest that MDSCs may play a role in regulating alloimmunity during GVHD.

Retinoic acid (RA), the active metabolite of vitamin A, has been implicated in GVHD pathogenesis in animal models (38, 39). Administration of exogenous RA exacerbates gut damage in recipient mice and significantly increases GVHD-associated mortality (31, 38, 39). We also showed the involvement of RA signaling in host APCs and intestinal epithelial cells in the pathophysiology of GVHD (40–42). However, whether RA signaling can act on donor myeloid cells to regulate alloimmune response remains unclear. It is of particular relevance to investigate the potential effects of RA on myeloid immune reconstitution after allogeneic HSCT since RA is well known for its strong effects on the proliferation and differentiation of immature myeloid cells (43, 44). Indeed, *all-trans* RA has been used successfully in the clinic to treat patients with acute promyelocytic leukemia due to its unique ability to induce the terminal differentiation of myeloid leukemic blasts (45, 46).

We hypothesized that RA signaling affects the reconstitution of some donor myeloid cell populations after allogeneic HSCT. In this study, we used a well-established mouse model of GVHD to test this hypothesis, focusing on donor intestinal CD103^+^ DCs and MDSCs. We showed that RA has the potential to influence post-transplant induction of these cells from donor BM progenitor cells.

## Materials and methods

### Mice

C57BL/6 (B6; H-2^b^) and Balb/c (H-2^d^) mice were purchased from The Jackson Laboratories (Bar Harbor, ME) or bred in the Biomedical Resource Center (BRC) at the Medical College of Wisconsin (MCW). For the experimental purpose of dietary modification of vitamin A levels, pregnant female Balb/c mice were fed a vitamin A-deficient (VAD) diet or a vitamin A-normal (VAN) diet (Envigo, Indianapolis, IN) from day 14.5 of gestation and maintained on the special diets until weaning of the litter. After weaning, litters were fed the same VAD or VAN food for 10-12 weeks prior to being used in experiments. All animals were housed in the Association for Assessment and Accreditation of Laboratory Animal Care (AAALAC)–accredited BRC of the MCW. All experiments were carried out under protocols approved by the MCW Institutional Animal Care and Use Committee.

### Murine model of BMT

BMT was performed as described previously (37). Briefly, BM was flushed from femurs and tibias of donor mice with Dulbecco′s Modified Eagle′s Medium (DMEM) and passed through sterile mesh filters to obtain single-cell suspensions. Splenic pan-T cells were purified from donor mice using EasySep™ Mouse T Cell Isolation Kit (StemCell Technologies, Cambridge, MA) according to the manufacturer’s instructions. Balb/c recipients were conditioned with total body irradiation administered as a single exposure of 900 Rads using a Shepherd Mark I Cesium Irradiator (J. L. Shepherd and Associates, San Fernando, CA). Irradiated recipients received a single intravenous injection of BM cells (6-10 x 10^6^) plus purified splenic pan-T cells (0.3-0.5 x 10^6^) in the lateral tail vein. In some experiments, exogenous RA was given to recipient mice via intraperitoneal injection after BMT. Some experiments were performed at the Basic Medical College of Harbin Medical University (Harbin, China). Specifically, lethally irradiated Balb/c mice (a single exposure of 7Gy using a Rad Source RS2000 X-ray irradiator) were transplanted with a mixture of BM cells (8 x 10^6^) and purified splenic pan-T cells (0.3 x 10^6^) from B6 donors. The RA dosing regimen was selected based on previously established *in vivo* protocols demonstrating immunomodulatory activity of exogenous RA (38, 39, 47, 48). For survival studies, recipient mice were weighed twice weekly and assessed weekly for clinical signs of GVHD. Clinical GVHD scores were based on five parameters: weight loss, posture, activity, fur texture, and skin integrity. Each parameter was scored from 0 to 2, for a maximal composite score of 10. Mice reaching predefined humane endpoints, including a moribund state or more than 30% body weight loss, were euthanized and recorded as mortality events for survival analysis.

### Histopathological evaluation of GVHD

Recipient mice were euthanized on day 14 after allogeneic BMT, and the small intestine, large intestine, liver, skin and lung were collected, fixed, and processed for hematoxylin and eosin (H&E) staining. Histopathological damage was evaluated by a pathologist blinded to the treatment groups using an established organ-specific scoring system adapted from published criteria (49–51). Individual histopathologic parameters, including villous blunting, crypt regeneration, surface erosion, ulceration, and inflammatory infiltrates in the intestine; portal tract expansion, bile duct damage, and hepatocellular injury in the liver; dermal fibrosis, fat loss, and epidermal hyperplasia in the skin; perivascular, peribranchial, and parenchymal inflammation in the lung, were scored on a scale of 0-3. Scores for individual parameters were summed to generate a total histopathological score for each organ, with higher scores indicating more severe GVHD-associated tissue injury.

### Flow cytometry and antibodies

Immune cells were labeled with monoclonal antibodies conjugated to fluorescein isothiocyanate (FITC), phycoerythrin (PE), allophycocyanin (APC), APC-Cy7, PE-Cy7, RB705, BV510, PerCP-Cy5.5 or PerCP-eFluor 710. PE-anti-CD4 (clone GK1.5), FITC-anti-CD8a (clone 53-6.7), PE-anti-TCRβ (clone H57-597), FITC-anti-H-2K^b^ (clone AF6-88.5), RB705-anti-H-2K^b^ (clone AF6-88.5), BV510-anti-H-2K^b^ (clone AF6-88.5), PE-Ly6G (clone 1A8), PE-CD103 (clone M290), FITC-Gr-1 (clone RB6-8C5), PE-Gr-1 (clone RB6-8C5), APC-Cy7-F4-80 (clone T45-2342), BV510-anti-CD84 (clone 1D3), APC-CD11c (clone HL3) were purchased from BD Biosciences. APC-Ly-6C (clone HK1.4), APC-Cy7-CD11b (clone 3A33), PerCP-eFluor 710-MHC-II (clone M5/114.15.2) were obtained from Invitrogen. APC-Cy7-CD103 (clone 2E7), PE-Cy7-CD11c (clone N418), APC-anti-XCR1(clone ZET) and PerCP-Cy5.5-CD172a (clone P84) were purchased from Biolegend. FITC-CD11b (clone M1/70) was obtained from eBioscience. Samples were analyzed using BD LSRII or Canto flow cytometer. Cell sorting was performed using a Beckman MoFlo XDP cell sorter. Data were analyzed using FlowJo software (TreeStar, Ashland, OR).

### Mixed lymphocyte reaction

Lethally irradiated Balb/c mice were transplanted with BM (8 x 10^6^) plus purified T cells (0.3 x 10^6^) from B6 mice. The recipients were then treated with DMSO or RA (400 μg) every other day by intraperitoneal injections. On day 7 post-BMT, mice were euthanized, and MDSCs were isolated from the spleens of DMSO- or RA-treated allogeneic BMT recipient mice using CD11b microbeads (Miltenyi Biotec, Bergisch Gladbach, Germany). Highly purified pan T cells (1 x 10^5^ cells/well) from the spleen of naïve B6 mice were labeled with Cell Trace Violet (Invitrogen, Waltham, MA) according to the manufacturer’s instructions. They were co-cultured with 5 x 10^4^ Balb/c DCs in 96 well U-bottomed plates at 37 °C in a complete RPMI medium containing 10% FBS. Graded numbers of MDSCs were added to some cultures. T cell proliferation was assessed by dye dilution using an LSRII flow cytometer after 4 days of culture. The division index (DI) was obtained by using Flowjo proliferation mode and % Suppression was calculated as described (52).

BM cells from B6 mice were cultured in the presence of GM-CSF (20 ng/ml) to generate DCs. DMSO or RA (100 nM) was added to some cultures at day 0. On day 7, DCs (5 x 10^4^) were isolated using CD11c microbeads (Miltenyi Biotec) and cocultured with carboxyfluorescein succinimidyl ester (CFSE, Invitrogen, Waltham, MA)-labeled pan-T cells (1 x 10^5^) from FVB mice in 96 well U-bottomed plates at 37 °C in a complete RPMI medium containing 10% FBS. T cell proliferation was assessed by dye dilution using an LSRII flow cytometer after 4 days of culture.

### Lamina propria dissociation

Colonic lamina propria cells were isolated with Lamina Propria Dissociation Kit (Miltenyi Biotec) according to the manufacturer’s instructions. Briefly, colons were cleared of surrounding fat and fecal contents, opened longitudinally, and cut into approximately 0.5cm fragments. Following pre-digestion to eliminate epithelial cells, tissues were enzymatically digested and mechanically dissociated using a gentleMACS Dissociator (Miltenyi Biotec). The resulting cell suspensions were passed through a 100μm cell strainer, pelleted by centrifugation, and resuspended for flow cytometric analysis.

### Bulk RNA-sequencing

Donor-derived MDSCs were obtained from the spleens of DMSO- or RA-treated recipient mice on day 7 post allogeneic BMT by flow cytometry-based cell sorting. The mRNA was isolated from total RNA by oligo-dT magnetic beads and then used to construct a sequencing library. The concentration of the library was quantified using a Qubit fluorometer, and the fragment size distribution was assessed using an Agilent Bioanalyzer. The library was sequenced on the Illumina NovaSeq 6000 platform with paired-end 150 (PE150) reads. The RNA-seq analyses were performed in R. DESeq2 was employed for differential expressed genes (DEGs) analysis. Genes with a padj (adjusted p-value) < 0.05 and | log2(fold change) | > 1 were considered significantly differentially expressed.

### Statistics

Data analysis was performed using Prism software (GraphPad, La Jolla, CA). Group comparisons were performed using an unpaired two-tailed Student’s *t*-test or one-way ANOVA. Survival curves were compared using the Log-rank (Mantel-Cox) test. Body weight percentages and GVHD clinical scores were compared between groups using the nonparametric Mann-Whitney U test. A *p* value < 0.05 was considered as statistical significance in all experiments.

## Results

### Expansion of donor derived MDSCs early after allogeneic BMT

We first examined whether MDSCs were detectable in recipient mice of allogeneic BMT. Lethally irradiated Balb/c mice were transplanted with MHC-mismatched B6 BM and purified splenic T cells to induce GVHD. Donor derived MDSCs (H-2K^b+^Gr1^+^CD11b^+^) were found to constitute about 40% of splenocytes at day 7 post-BMT and the percentage gradually declined thereafter. In contrast, only 3∼4% of such Gr1^+^CD11b^+^ cells were detected in the spleen of naïve Balb/c mice (**Figures 1A–C**). These results are consistent with our previous studies (31) and indicate that there was a significant expansion of MDSCs in the spleens of recipient mice after allogenic BMT.

**Figure 1:**
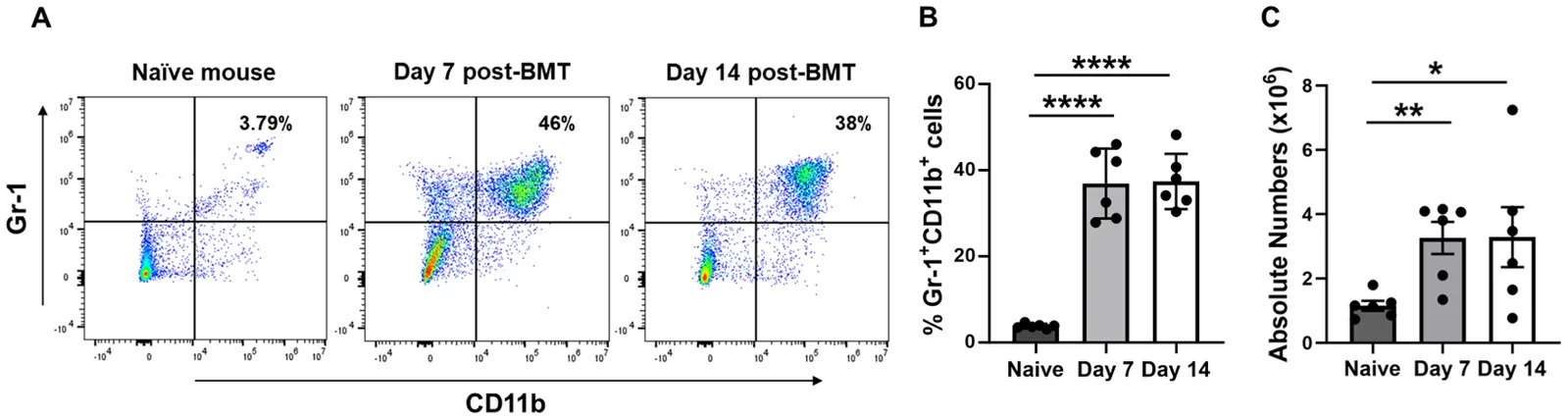
Expansion of donor derived MDSCs early after allogeneic BMT. Lethally irradiated Balb/c mice received a transplant of BM (8 x 10^6^) and spleen cells (0.5 x 10^6^) from B6 mice. Recipient mice were euthanized at day 7 and day 14 after BMT. The percentage of donor derived MDSCs (H-2K^b+^Gr1^+^CD11b^+^) in the spleen of recipient mice was determined by flow cytometry. **(A)** Representative flow plots depicting Gr1 and CD11b expression on splenocytes are shown. Mean (± SD) percentage **(B)** and absolute number **(C)** of H-2K^b+^Gr1^+^CD11b^+^ cells in the spleen of recipient mice. Data are cumulative results from two independent experiments (n=6 per group). Statistics: *P<0.05, **P<0.01, and ****P<0.0001.

### RA regulates the accumulation of MDSCs after allogeneic BMT

We then studied whether RA signaling participates in regulating the accumulation and function of this cell population. To address this question, lethally irradiated Balb/c mice were transplanted with BM and purified T cells from B6 mice. Recipient mice were then treated with either DMSO or RA. We found a significant reduction in the absolute number of donor-derived Gr1^+^CD11b^+^ MDSCs in the spleen of RA-treated mice compared to DMSO-treated mice, although there was no significant difference in the percentage of these cells between the two groups (**Supplementary Figure 1A**). There were also no significant differences in either the percentage or absolute number of donor Gr1^+^CD11b^+^ MDSCs in the BM of recipient mice between the two groups (**Supplementary Figure 1B**). To identify MDSCs more stringently, F4-80 (53) and CD84 (54) were introduced as additional surface markers (**Figures 2A, B**). Consistent with previous results, we observed a significant reduction in the absolute number of F4-80^-^CD84^high^Gr1^+^CD11b^+^ MDSCs in the spleen of RA-treated mice compared to DMSO-treated mice (**Figure 2C**). Importantly, we found that MDSCs isolated from RA-treated mice showed significantly decreased ability to suppress the proliferation of alloreactive T cells compared to those from DMSO-treated mice (**Figures 2D, E**). Consistent results were obtained from 3 different effector T-cell to MDSC ratios tested. Thus, MDSCs expand in recipient mice after allogeneic BMT and exogenous RA causes both quantitative and qualitative changes of these cells, which may affect the expansion of donor T cells after allogeneic BMT. These data indicate the involvement of RA signaling in the differentiation and function of MDSCs after BMT.

**Figure 2:**
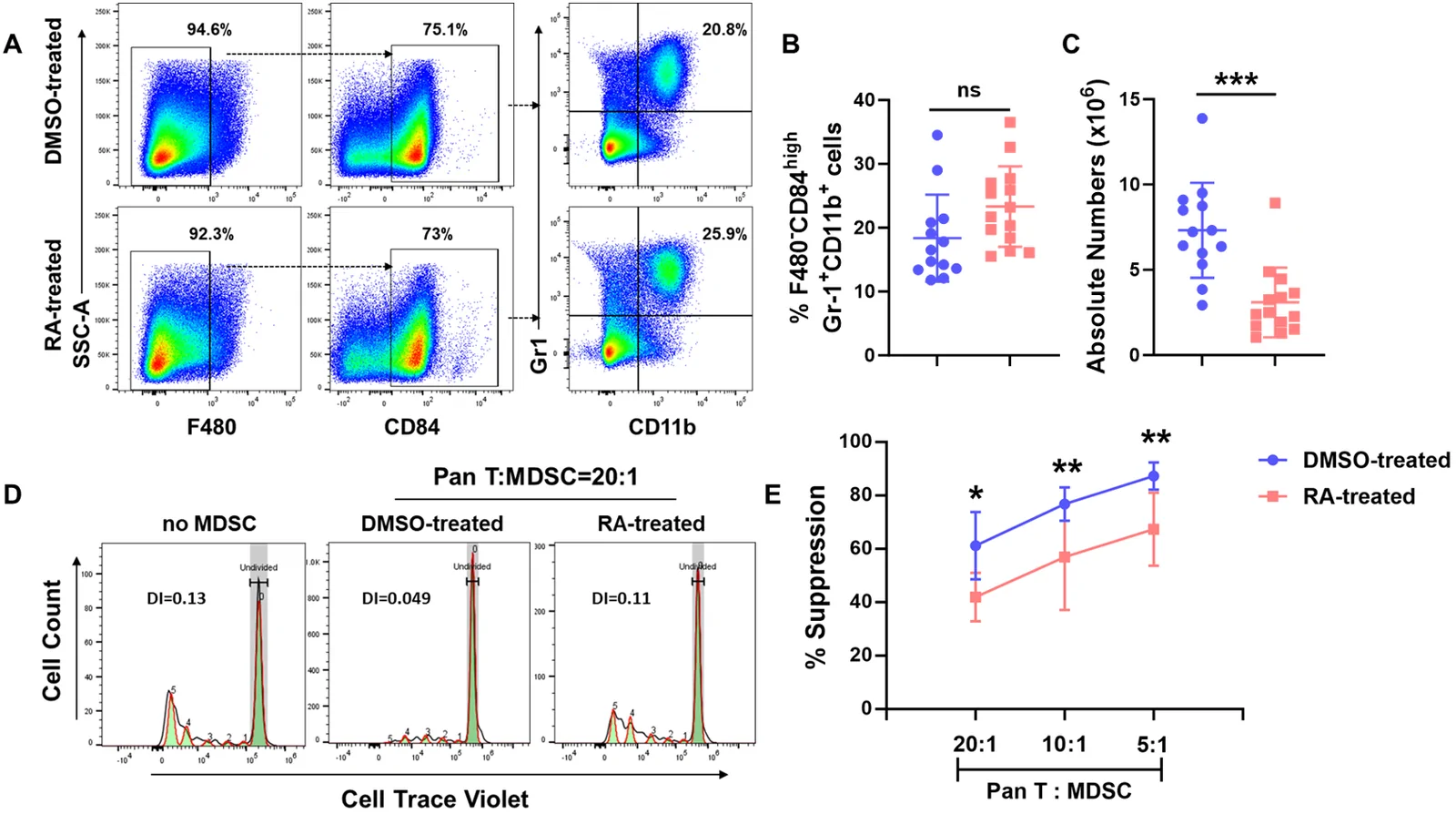
RA treatment causes quantitative and qualitative defects of MDSCs after BMT. Lethally irradiated Balb/c mice were transplanted with BM (7–8 x 10^6^) plus purified T cells (0.3 x 10^6^) from B6 mice. The recipients were then treated with DMSO or RA (400 μg) every other day by intraperitoneal injections. On day 7 after BMT, the mice were euthanized. The percentage **(A)** and absolute number **(B)** of Gr1^+^CD11b^+^CD84^+^F4-80^-^ cells in the spleen were examined. Data are shown as Mean ± SD and are cumulative results from 3 independent experiments (n=13-14 per group). **(C)** Cell trace-Violet labeled pan-T cells (1 x 10^5^) from B6 mice were cocultured with DCs (5 x 10^4^) from Balb/c mice to set up MLRs. Different numbers of MDSCs from DMSO or RA-treated allogeneic BMT mice were added to some culture wells. The proliferation of T cells was determined by flow cytometry after 4 days. Data are shown as Mean (± SD) percentage of suppression of alloreactive CD4^+^ T cells. %Suppression=100-(DI1/DI2)100. Division Index 1 (DI1) denotes T cells cocultured with DCs and MDSCs, Division Index 2 (DI2) denotes T cells cocultured with DCs without MDSCs. Data are cumulative results from two independent experiments. Statistics: *P<0.05, **P<0.01, and ***P<0.001.

### Wnt signaling and the CCL2-CCR2 axis are involved in the observed effects of RA on MDSCs

To explore the potential mechanism by which RA modulates MDSCs after allogeneic BMT, we isolated donor-derived F4-80^-^CD84^high^Gr1^+^CD11b^+^ MDSCs from the spleens of recipients for bulk RNA-seq. Enrichment analyses of DEGs (**Figure 3A**) were performed using GO (**Supplementary Figures 2A, B**) and KEGG databases (**Supplementary Figures 2C, D**). Analysis of DEGs revealed that RA significantly reduced CCR2 levels (**Figure 3B**). Gene Set Enrichment Analysis (GSEA) results showed that RA treatment decreased the transcription of genes associated with DNA replication (R-HSA-69306) (**Figure 3C**; **Supplementary Figure 2E**) and S phase (R-HAS-69242) (**Figure 3D**; **Supplementary Figure 2F**), and increased the transcription of genes associated with negative regulation of Wnt signaling pathway (GO:0030178) (**Figure 3E**; **Supplementary Figure 2G**). Previous studies have reported that disrupting the CCL2-CCR2 pathway can impair MDSC chemotaxis and suppressive function (55, 56), while Wnt pathway activation promotes the recruitment, viability and function of MDSC (57) and upregulates CCL2-CCR2 axis (58). These results showed that RA treatment downregulated CCR2 expression and enriched gene signatures associated with negative regulation of Wnt signaling in MDSCs in our model.

**Figure 3:**
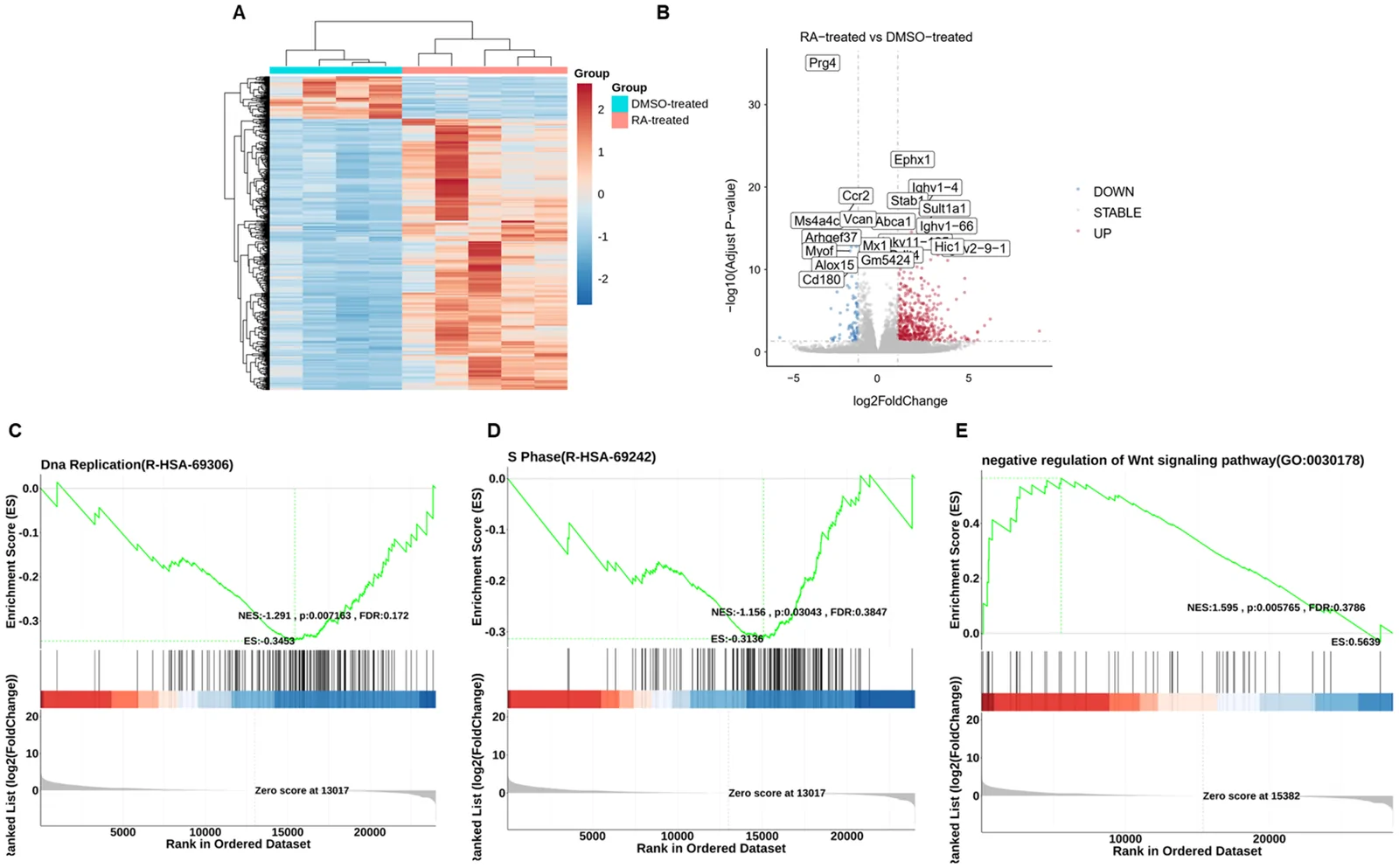
Bulk RNA-seq of donor-derived MDSCs. Lethally irradiated Balb/c mice were transplanted with BM (8 x 10^6^) plus purified T cells (0.3 x 10^6^) from B6 mice. The recipients were then treated with DMSO or RA (400 μg) every other day by intraperitoneal injections. On day 7 after BMT, the mice were euthanized and highly purified donor-derived MDSCs (F4-80^-^CD84^high^Gr1^+^CD11b^+^) were obtained by cell sorting for bulk RNA-seq. **(A)** The clustering diagram of DEGs was shown. **(B)** Volcano plot presented the DEGs in donor-derived MDSCs from DMSO- or RA-treated recipients. **(C–E)** GESA was performed in those genes that were significantly different in donor-derived MDSCs from DMSO- or RA-treated recipients. Genes with a padj (adjusted p-value) < 0.05 were considered differentially expressed. Genes with a padj (adjusted p-value) < 0.05 and | log2(fold change) | > 1 were defined as DEGs.

### RA promotes the induction of granulocytic MDSCs

In mice, two types of MDSCs have been defined: monocytic MDSCs (M-MDSCs) and granulocytic/polymorphonuclear MDSCs (G-MDSCs). Phenotypically, M-MDSCs are defined as CD11b^+^Ly6G^-^Ly6C^high^ while G-MDSCs are CD11b^+^Ly6G^+^Ly6C^low^ (15). It is worth mentioning that the anti-Gr-1 antibody (clone RB6-8C5) recognizes both Ly6G and Ly6C, whereas the anti-Ly6G antibody (clone 1A8) and the anti-Ly6C antibody (clone HK1.4) recognize Ly6G and Ly6C, respectively. To investigate how RA affects the differentiation of MDSCs from BM precursors, we used a well-established *in vitro* culture system (59). In the presence of cytokines GM-CSF and IL-6, BM cells can differentiate into both M-MDSCs and G-MDSCs. Interestingly, the addition of RA to cultures skewed the differentiation of BM progenitors toward CD11b^+^Ly6G^+^Ly6C^low^ G-MDSCs (**Figures 4A, B**). On the contrary, in the presence of a pan-RAR inhibitor, there was a significantly higher percentage of M-MDSCs in the culture. These results showed that RA signaling affects the composition of different MDSC populations differentiated from BM precursor cells.

**Figure 4:**
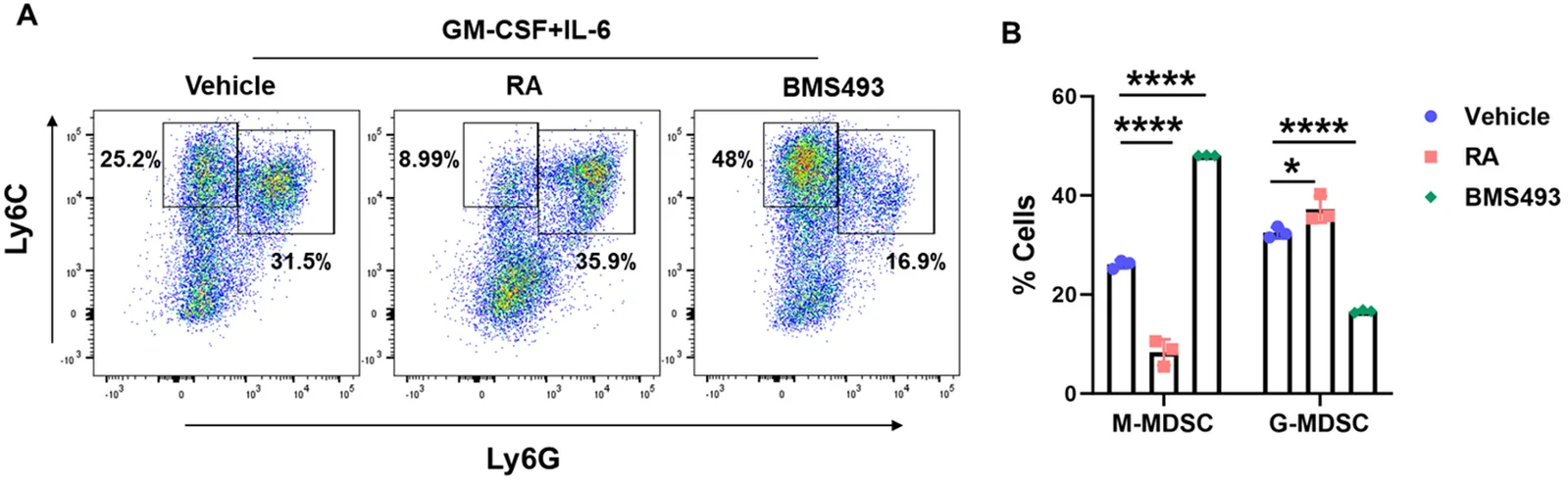
RA promotes the induction of granulocytic MDSCs *in vitro*. BM cells (0.75x10^6^/ml) from B6 mice were cultured in 6-well plates in the presence of GM-CSF (40 ng/ml) and IL-6 (40 ng/ml). RA (100 nM) or RA signaling inhibitor BMS493 (1 uM) was added to some cultures at day 0. The frequency of monocytic MDSCs (CD11b^+^Ly6C^+^Ly6G^-^) and granulocytic MDSCs (CD11b^+^Ly6C^-^ Ly6G^+^) MDSCs was evaluated after 3 days of culture by flow cytometry.​​ **(A)** Representative flow plots are shown; **(B)** Mean (± SD) percentage of M-MDSCs and G-MDSCs under different culture conditions. Data are derived from one of the two independent experiments. Statistics: *P<0.05 and ****P<0.0001.

### RA exacerbates GVHD and promotes colonic CD103+ DC reconstitution after allogeneic BMT

Consistent with previous studies, administration of exogenous RA exacerbated GVHD, as demonstrated by greater weight loss and higher GVHD clinical scores (**Figures 5A, B**). RA treatment also significantly reduced recipient survival (**Figure 5C**) and increased intestinal histopathological damage (**Figures 5D, E**). Importantly, recent studies have identified donor colonic CD103^+^ DCs as an important cell population that can amplify GVHD severity, especially in the GI tract (9, 10). We thus hypothesized that RA promotes the differentiation of intestinal CD103^+^ DCs from infused donor BM cells. To test this hypothesis, we set up allogeneic BMT and treated recipient mice with either DMSO or RA. On day 14 after transplantation, there was a significant increase in the percentage (**Figures 6A, B**) and absolute number (**Figure 6C**) of donor-derived CD103^+^ DCs in the colon of RA-treated mice compared to DMSO-treated mice, indicating that RA facilitates the reconstitution of donor CD103^+^ DCs after BMT. Interestingly, these CD103^+^ DCs were predominantly XCR1^+^ conventional DC1 in both groups (**Supplementary Figure 3**). To further confirm the involvement of RA, we generated vitamin A-deficient Blab/c mice and used them as recipients in our GVHD model. We found a significant reduction in the percentage (**Figures 6D, E**) and absolute number (**Figure 6F**) of CD11c^+^MHC-II^high^CD11b^-^CD103^+^ DC population in VAD mice compared to vitamin A normal mice. Collectively, these data demonstrated that RA signaling facilitates the generation of donor CD103^+^ intestinal DCs that have been shown to play a critical role in propagating GVHD.

**Figure 5:**
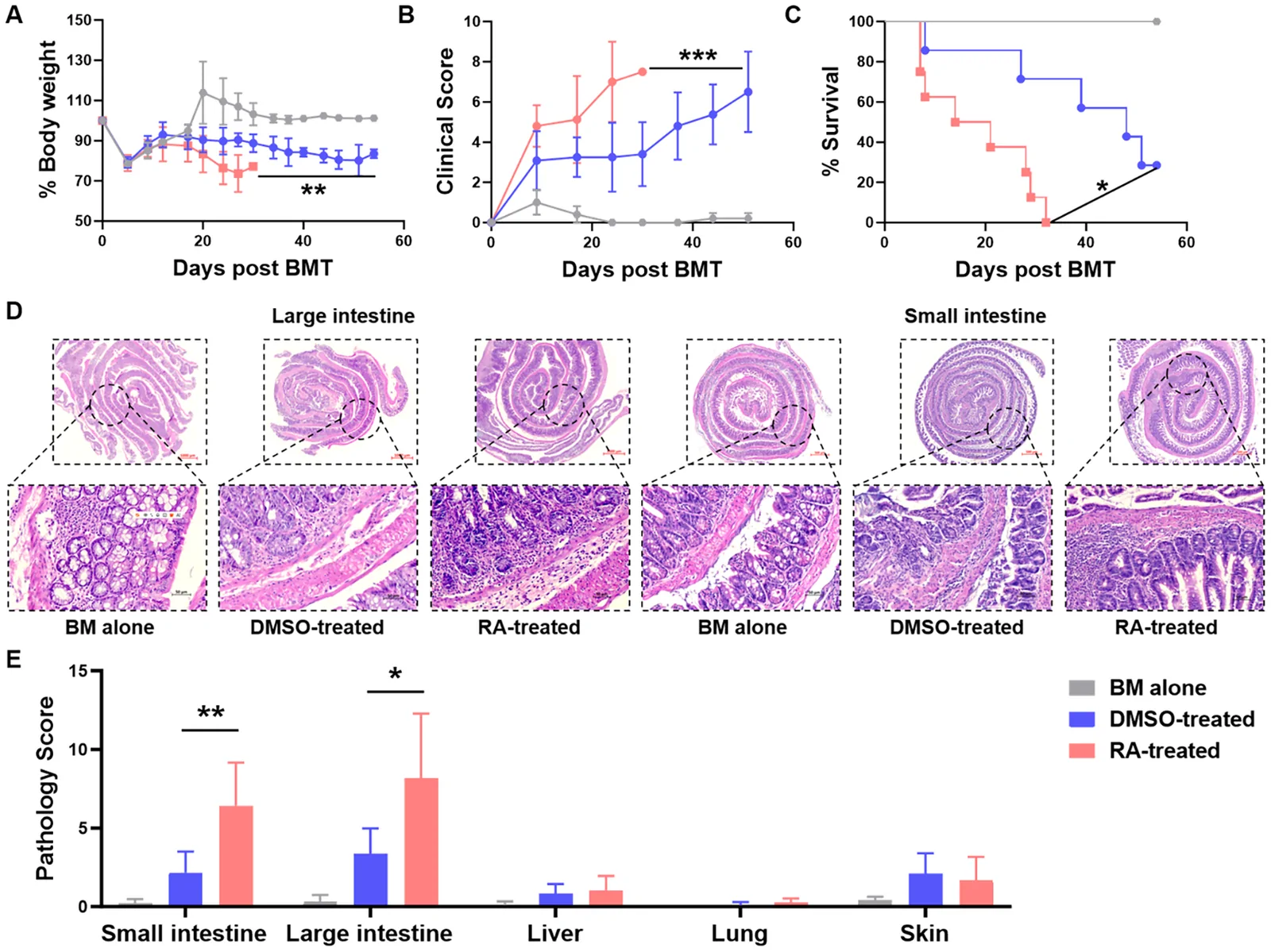
Exogenous RA treatment exacerbates GVHD. Lethally irradiated Balb/c mice were transplanted with BM (8 x 10^6^) alone or plus purified T cells (0.3 x 10^6^) from B6 mice. Mice receiving BM plus T cells were then treated with DMSO or RA (400 μg) every other day by intraperitoneal injections for two weeks. Mice were monitored for **(A)** body weight loss, **(B)** clinical GVHD scores and **(C)** survival (n=5-8 per group). In parallel experiments using the same allogeneic BMT model, recipient tissues were harvested on day 14 after BMT for histopathological analysis. **(D)** Representative H&E-stained sections of the small and large intestines. **(E)** Histopathological scores of GVHD target organs (n=5-6 per group). Data are shown as Mean (± SD) for body weight loss, clinical GVHD scores and histopathological scores. Statistics: *P<0.05, **P<0.01, ***P<0.001.

**Figure 6:**
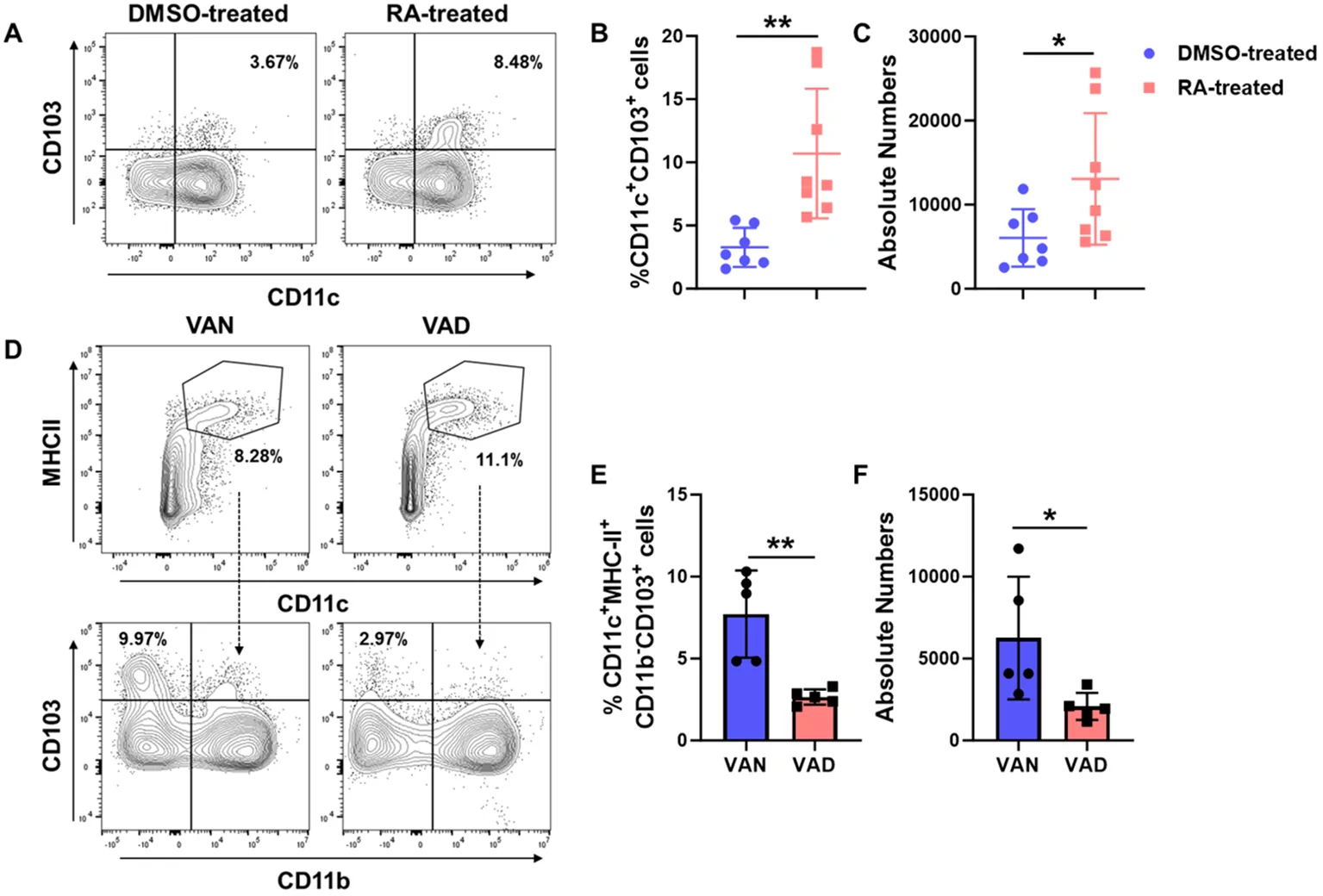
RA signaling promotes the induction of CD103^+^ DCs after BMT. Lethally irradiated Balb/c mice were transplanted with BM and purified T cells from B6 mice. They were then treated with either DMSO or RA (400 μg). On day 14 after transplantation, colon tissues were harvested. **(A)** The frequency of CD11c^+^CD103^+^ DCs was examined by flow cytometry and **(B)** the absolute number was calculated. Data are shown as Mean (± SD) and are cumulative results from two independent experiments (n=7-8 per group). Lethally irradiated vitamin A normal (VAN) or vitamin A deficient (VAD) Balb/c mice were transplanted with BM and purified T cells from B6 mice. Two weeks after BMT, recipient mice were euthanized and colon tissues were harvested. The frequency **(C, D)** and the absolute number **(E)** of CD11c^+^MHC-II^+^CD11b^-^CD103^+^ DCs were determined by flow cytometry and cell counting. Data are shown as Mean (± SD) and are cumulative results from two independent experiments (n=5 per group). Statistics: *P<0.05, **P<0.01.

### RA increases the induction of CD103^+^ DCs *in vitro*

We then investigated how RA affects the induction of CD103^+^ DCs in a culture system. DCs were generated from B6 BM cells in the presence of GM-CSF. There was a significant increase in the percentage of CD11c^+^CD103^+^ DCs in cultures containing RA, demonstrating that RA signaling promotes the differentiation of this unique DC subset from BM precursors (**Figure 7A**). On the contrary, the percentage of CD103^+^CD11c^+^ DCs was substantially decreased in cultures containing BMS493, a pan-RAR antagonist (**Figure 7B**), further reinforcing the involvement of RA signaling in the induction of this DC subset. Interestingly, RA also increased the expression of MHC-II on DCs (**Figure 7C**). BM-derived DCs exposed to RA induced a significantly greater proliferation of alloreactive CD4^+^ (**Figures 7D, E**) and CD8^+^ T cells (**Figures 7F, G**) compared with DMSO-treated DCs. These results suggested that RA promotes the generation of CD103^+^ DCs with enhanced T cell-stimulatory capacity.

**Figure 7:**
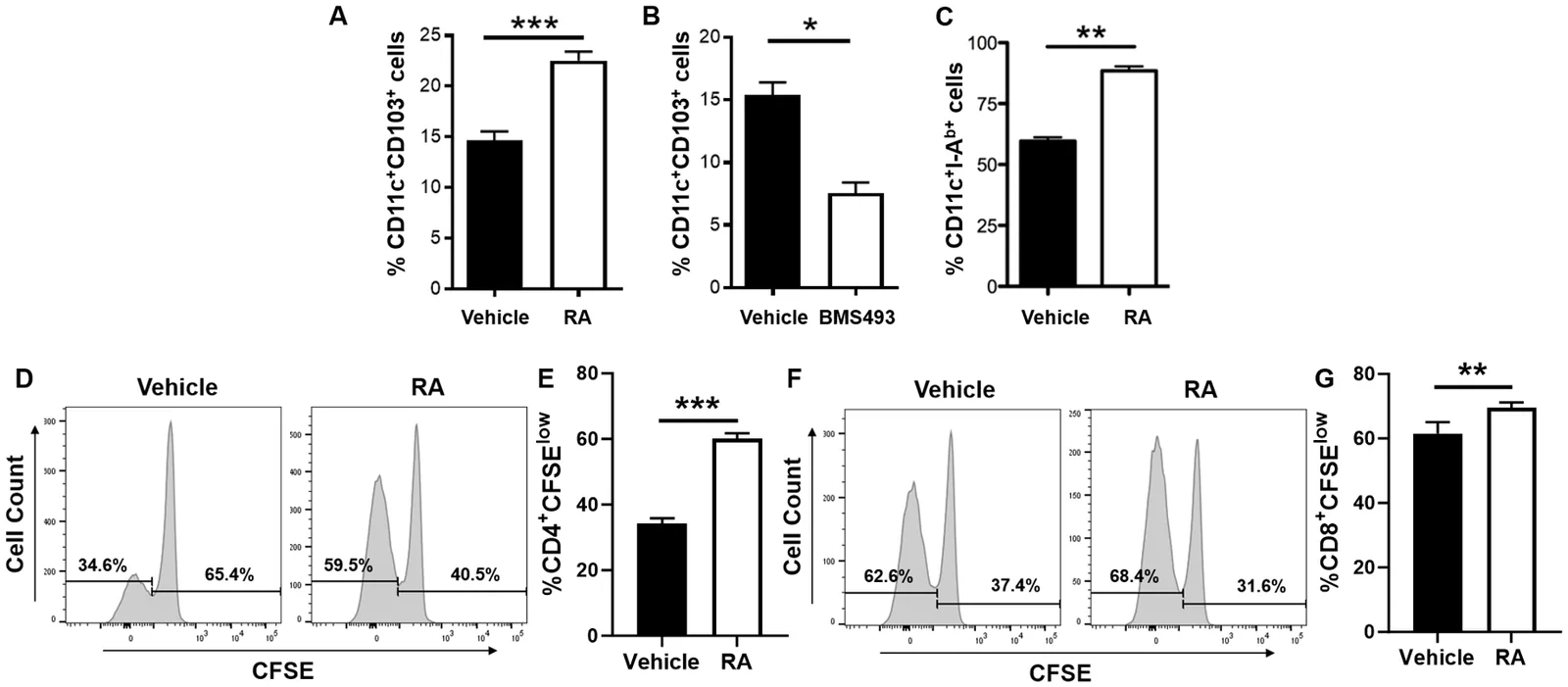
RA increases the induction of CD103^+^ DCs *in vitro*. BM cells from B6 mice were cultured in the presence of GM-CSF (20 ng/ml) to generate DCs. RA (100 nM) or BMS493 (1 uM) was added to some cultures at day 0. The expression of CD103 **(A, B)** and MHC-II **(C)** on DCs was assessed by flow cytometry on day 7. DMSO and RA-treated DCs (5 x 104) were further isolated and cocultured with CFSE-labeled pan-T cells (1 x 10^5^) from FVB mice to set up MLRs. The proliferation of T cells was determined by flow cytometry after 4 days. Data are shown as Mean (± SD). Data for RA-treated DCs are cumulative results from two to four independent experiments and data for BMS-493-treated DCs are cumulative results from two independent experiments. MLR data are derived from one of the two independent experiments. Statistics: *P<0.05, **P<0.01 and ***P<0.001.

## Discussion

GVHD is a donor T-cell mediated inflammatory disease. However, several myeloid cell populations of either host or donor origin have been shown to actively participate in the pathophysiology of GVHD. The goal of this study was to examine how RA signaling, which is well-known for its effects on myeloid cell differentiation and function, may regulate alloimmune response through modulating donor MDCSs and intestinal CD103^+^ DCs. We focused on these two cell types because of their myeloid origin and involvement in GVHD pathogenesis.

MDSCs are defined as heterogenous populations of immature myeloid cells with potent immunosuppressive function. The abnormal accumulation of MDSCs occurs under several pathologic conditions associated with chronic inflammation, infections, or physical stress (14, 15). After allogeneic BMT, donor BM progenitor cells repopulate the recipient’s hematopoietic system. We reasoned that a highly inflammatory environment in BMT recipients would disturb the normal differentiation of myeloid cells from infused hematopoietic stem/progenitor cells. Indeed, we and others observed an abnormal expansion of donor derived MDSCs early after allogeneic BMT. This phenomenon is likely due to increased levels of proinflammatory cytokines known to cause MDSC expansion, including GM-CSF and IL-6 (60, 61). Results from this paper showed that the RA pathway may influence both the quantity and quality of MDSCs after allogeneic BMT. We showed that RA treatment not only reduced the absolute number of MDSCs but also decreased their immunosuppressive function. This is consistent with the observation that RA can induce the terminal differentiation of MDSCs and reverse their suppression of antitumor immunity in tumor bearing hosts (62, 63). Interestingly, when RA was added to cultures, it skewed the differentiation of BM progenitors toward Ly6G^+^Ly6C^-^ granulocytic MDSCs. It is generally accepted that granulocytic MDSCs are less immunosuppressive than monocytic MDSCs on a per-cell basis (15, 64, 65). Thus, it is possible that recipient RA levels may affect the composition and function of MDSCs in recipients of allogeneic BMT. CCR2 has been identified as a key mediator of MDSC recruitment and maintenance within lymphoid tissues. Genetic or pharmacological inhibition of CCR2 results in blockade of radiation-induced MDSC infiltration and immunosuppression (55, 56). Downregulation of CCR2 expression, as revealed by the Bulk RNA-seq, may contribute to the diminished accumulation of MDSCs following RA treatment. In parallel, suppression of Wnt-associated transcriptional programs may disrupt pathways required for maintaining immature, suppressive myeloid states, leading to impaired MDSC function. Together, these transcriptional changes suggest potential mechanisms linking RA treatment to quantitative and functional alterations in MDSCs in our model. However, RNA-seq findings, including differential expression of Wnt pathway-related genes and CCR2 in RA-exposed MDSCs, were not independently validated using additional molecular or protein-based assays. In addition, functional studies were not performed to establish a causal role for these pathways. Further studies will therefore be required to validate these transcriptomic findings and determine whether Wnt signaling and CCR2 contribute mechanistically to RA-dependent changes in MDSC accumulation and suppressive function.

While pharmacologic approaches have long been the mainstay of GVHD prevention and treatment, recent studies have demonstrated the clinical value of using immunosuppressive cells to control GVHD (66, 67). Adoptive transfer of *in vitro* generated MDSCs has been shown to mitigate GVHD in animal models. An increase in MDSC number and functionality may inhibit the expansion of alloreactive donor T cells after HSCT. Whether pharmacologic modulation of RA signaling is feasible and safe in clinical HSCT requires further study given the pleiotropic effects of RA *in vivo*. The net effect of RA signaling modulation may vary by cell type, timing, and dose. However, our RNA-seq data implicate altered Wnt signaling and CCL2-CCR2 axis in RA-associated MDSC dysregulation, suggesting that therapeutic modulation of downstream pathways may represent an alternative strategy to influence alloimmune responses after transplantation. Modulating the composition and function of MDSCs to enhance their immunosuppressive properties *in vivo* may restrain donor T cell expansion and reduce GVHD.

Nevertheless, the present study was conducted using a single murine transplant strain combination, and the effects of RA on MDSCs were not evaluated in human cells. Future studies using additional murine transplant models, as well as human or humanized experimental systems, will be important to determine the generalizability and translational relevance of our findings.

RA has been shown to facilitate the differentiation of dendritic cells from myeloid precursors under certain culture conditions (68, 69). Mucosal CD103^+^ DCs in the gut-associated lymphoid tissues are critical for maintaining oral tolerance and intestinal homeostasis under steady-state conditions due to their unique ability to produce RA and induce the peripheral conversion of naïve T cells into iTregs (70, 71). RA plays an important role in the development and homeostasis of intestinal CD103^+^ DCs. RA/RAR-α signaling promotes the generation of gut-homing DC precursors in the bone marrow that preferentially give rise to intestinal CD103^+^ DCs (72). RA also regulates the homeostasis and differentiation of intestinal CD103^+^ DC subsets and contributes to their transcriptional programming and specialization (73, 74). However, during intestinal inflammation, CD103^+^ DCs can lose their tolerogenic potential and instead promote colitis (75). Thus, CD103^+^ mucosal DCs may play a detrimental role under pathogenic conditions. During inflammation, such as a period of immune reconstitution after allogenic BMT, strong innate immune signals and inflammatory cytokines such as IL-6, Il-12, and IL-23 reprogram RA signaling toward effector differentiation, allowing it to support Th1 and Th17 responses and enhance T cell survival and function (76, 77). Thus, RA does not intrinsically dictate tolerance or inflammation but instead amplifies the dominant immune context present in the tissue. Importantly, recent studies have identified donor colonic CD103^+^ DCs as a key cell population that can intensify GVHD, especially in the GI tract (9, 10). Specifically, CD103^+^CD11b^-^ DCs amplifies GVHD severity by their capacity of indirect alloantigen presentation, IL-12 production, and ability to enhance donor T cell proliferation and migration to the gut (9). Mucosal DCs are derived from BM pre-mucosal DC precursors and require RA (72, 73, 78–80). We found that exogenous RA exacerbated GVHD and increased the percentage and absolute number of colonic CD103^+^CD11b^-^ DCs during GVHD. On the contrary, vitamin A deficiency significantly reduced the percentage and absolute number of this cell population in recipient mice after allogeneic BMT. These results suggested that the level of RA signaling may influence the reconstitution of donor mucosal CD103^+^ DCs, a critical pathogenic cell population that determines GVHD severity after allogeneic BMT. We further showed that RA exposure increased the percentage of CD11c^+^CD103^+^ DCs with increased expression of MHC-II and T-cell stimulatory capacity in BM cultures. An increase in MHC-II expression on DCs can enhance the ability of these cells to present alloantigen to donor T cells. Based on these studies, we speculate that exogenous RA may similarly act on donor-derived DC precursors during immune reconstitution following allogeneic BMT. This may favor the development and/or maintenance of CD103^+^ DCs and contribute to their increased accumulation in the intestine. However, whether the increased CD103^+^ DC population reflects altered differentiation, survival, proliferation, or trafficking remains to be determined. It is of interest to investigate whether targeting RA signaling can reduce the reconstitution of intestinal CD103^+^ DCs and reduce GVHD. In addition, future studies examining the kinetics of donor-derived DC reconstitution will help define its temporal relationship with GVHD progression.

The differential effects of RA on MDSCs and colonic DCs may reflect its distinct effects on myeloid differentiation and tissue-specific reconstitution following allogeneic BMT. RA can promote the differentiation of immature myeloid cells, thereby limit MDSC accumulation, while support the differentiation and tissue adaptation of intestinal DCs. Thus, RA may shift post-transplant myeloid reconstitution away from immature suppressive MDSCs and toward more differentiated DC populations. Further studies are needed to define the mechanisms underlying this differential regulation. In summary, our results indicate that RA signaling may affect the reconstitution of some donor myeloid cell populations after allogeneic BMT. Specifically, RA alters MDSC composition and function, which is associated with changes in Wnt signaling and the CCL2-CCR2 axis. It also influences the differentiation of colonic CD103^+^ DCs. Given the importance of these cells in GVHD development, therapeutic modulation of RA signaling or its downstream pathways may represent a strategy to limit alloimmunity and improve transplant outcomes.

## Data Availability

The original contributions presented in the study are included in the article/**Supplementary Material**. Further inquiries can be directed to the corresponding author.
