# Supplementary material for: Retinoic acid regulates donor myeloid-derived suppressor cell and colonic dendrite cell reconstitution after allogeneic hematopoietic stem cell transplantation in mice
Source: Front Immunol. 2026 Sep 22;17:1599346. doi: 10.3389/fimmu.2026.1599346 (PMC13640452; doi:10.3389/fimmu.2026.1599346)
Supplement: Supplementary file 1 [file SupplementaryFile1.docx]

**Supplemental Figure 1. RA treatment reduces splenic MDSCs after BMT.** Lethally irradiated Balb/C mice were transplanted with BM (7–8 x 10^6^) plus purified T cells (0.3 x 10^6^) from B6 mice. The recipients were then treated with DMSO or RA (400 μg) every other day by intraperitoneal injections. On day 7 after BMT, the mice were euthanized. The percentage and absolute number of Gr-1^+^CD11b^+^ cells in the spleen **(A)** and BM **(B)** were examined. Data are shown as Mean ± SEM and are cumulative results from 3 independent experiments (n=6−8 per group). Statistics: **P<0.01.

**Supplemental Figure 2. Bulk RNA sequencing of donor-derived MDSCs.** Lethally irradiated Balb/C mice were transplanted with BM (8 x 10^6^) plus purified T cells (0.3 x 10^6^) from B6 mice. The recipients were then treated with DMSO or RA (400 μg) every other day by intraperitoneal injections. On day 7 after BMT, the mice were euthanized and highly purified donor-derived MDSCs (F4-80^-^CD84^high^Gr1^+^CD11b^+^) were obtained by cell sorting for bulk RNA sequencing. (**A**) GO enrichment analyses were performed on DEGs shown in Figure 3A. (**B**) DEGs in the top5 pathways of enriched GO BP, MF and CC terms were presented. (**C**) KEGG enrichment analyses were performed on DEGs shown in Figure 3A. (**D**) DEGs in the top5 pathways of enriched KEGG terms were presented. GESA was performed in those genes that were significantly different in donor-derived MDSCs from DMSO- or RA-treated recipients. Transcription diagram of (**E**) DNA replication (R-HSA-69306)-, (**F**) S phase (R-HSA-69242)- and (**G**) negative regulation of Wnt signaling pathway (GO:0030178)-related differential genes were shown.

**Supplemental Figure 3. Colonic CD103^+^ DCs after allogeneic BMT are primarily the cDC1 subset.**  Lethally irradiated Balb/c mice were transplanted with BM and purified T cells from B6 mice. They were then treated with either DMSO or RA (400 ug). On day 14 after transplantation, colon tissues were harvested. The expression of XCR1^+^ or CD172a^+^ on gated CD11c^+^CD103^+^ DCs was determined by flow cytometry. Data are shown as Mean (± SD) and are cumulative results from two independent experiments.
